# Effects of ertugliflozin on renal function over 104 weeks of treatment: a post hoc analysis of two randomised controlled trials

**DOI:** 10.1007/s00125-020-05133-4

**Published:** 2020-03-31

**Authors:** David Z. I. Cherney, Hiddo J. L. Heerspink, Robert Frederich, Mario Maldonado, Jie Liu, Annpey Pong, Zhi J. Xu, Shrita Patel, Anne Hickman, James P. Mancuso, Ira Gantz, Steven G. Terra

**Affiliations:** 1 Division of Nephrology, University of Toronto, Toronto General Hospital, 585 University Ave, 8N-845, Toronto, ON M5G 2N2 Canada; 2 The George Institute for Global Health, Sydney, NSW Australia; 3 Pfizer Inc., Collegeville, PA USA; 4 Merck Sharp & Dohme Limited, London, UK; 5 Merck & Co., Inc., Kenilworth, NJ USA; 6 Pfizer Inc., Groton, CT USA; 7 Pfizer Inc., Andover, MA USA

**Keywords:** Albuminuria, Diabetic nephropathies, Ertugliflozin, Glomerular filtration rate, Type 2 diabetes mellitus

## Abstract

**Aims/hypothesis:**

This study aimed to evaluate the effect of ertugliflozin, a sodium–glucose cotransporter 2 (SGLT2) inhibitor, on eGFR and albuminuria (urine albumin/creatinine ratio [UACR]) vs glimepiride or placebo/glimepiride (non-ertugliflozin) over 104 weeks of treatment in participants with type 2 diabetes mellitus, using pooled data from two randomised controlled, active comparator studies from the eValuation of ERTugliflozin effIcacy and Safety (VERTIS) programme (Clinicaltrials.gov NCT01999218 [VERTIS SU] and NCT02033889 [VERTIS MET]). In the VERTIS SU study, ertugliflozin was evaluated vs glimepiride over 104 weeks. In the VERTIS MET study, ertugliflozin was evaluated vs placebo over 26 weeks; eligible participants were switched from placebo to blinded glimepiride from week 26 to week 104. The glycaemic efficacy of ertugliflozin vs non-ertugliflozin was also assessed in the pooled population.

**Methods:**

Post hoc, exploratory analysis was used to investigate mean changes from baseline in eGFR and UACR over 104 weeks.

**Results:**

Overall, mean (SD) baseline eGFR was 88.2 (18.8) ml min^−1^ (1.73 m)^−2^ and geometric mean (95% CI) of baseline UACR was 1.31 mg/mmol (1.23, 1.38). At week 6, the changes in eGFR from baseline were −2.3, −2.7 and −0.7 ml min^−1^ (1.73 m)^−2^ for the ertugliflozin 5 mg, ertugliflozin 15 mg and non-ertugliflozin groups, respectively. Mean eGFR in the ertugliflozin groups increased over time thereafter, while it decreased in the non-ertugliflozin group. Week 104 changes in eGFR from baseline were −0.2, 0.1 and −2.0 ml min^−1^ (1.73 m)^−2^ for the ertugliflozin 5 mg, ertugliflozin 15 mg and non-ertugliflozin groups, respectively. Among 415 patients (21.4% of the cohort) with albuminuria at baseline, the ertugliflozin groups had greater reductions in UACR at all measured time points up to week 104. At week 104, the non-ertugliflozin-corrected difference in UACR (95% CI) was −29.5% (−44.8, −9.8; *p* < 0.01) for ertugliflozin 5 mg and −37.6% (−51.8, −19.2; *p* < 0.001) for ertugliflozin 15 mg. Least squares mean changes from baseline in HbA_1c_ (mmol/mol [95% CI]) at week 104 were similar between treatment groups: −6.84 (−7.64, −6.03), −7.74 (−8.54, −6.94) and −6.84 (−7.65, −6.03) in the ertugliflozin 5 mg, ertugliflozin 15 mg and non-ertugliflozin groups, respectively. Least squares mean changes from baseline in HbA1_c_ (% [95% CI]) at week 104 were: −0.63 (−0.70, −0.55), −0.71 (−0.78, −0.64) and −0.63 (−0.70, −0.55) in the ertugliflozin 5 mg, ertugliflozin 15 mg and non-ertugliflozin groups, respectively.

**Conclusions/interpretation:**

Ertugliflozin reduced eGFR at week 6, consistent with the known pharmacodynamic effects of SGLT2 inhibitors on renal function. Over 104 weeks, eGFR values returned to baseline and were higher with ertugliflozin compared with non-ertugliflozin treatment, even though changes in HbA_1c_ did not differ between the groups. Ertugliflozin reduced UACR in patients with baseline albuminuria.

**Trial registration:**

clinicaltrials.gov NCT01999218 and NCT02033889.

**Electronic supplementary material:**

The online version of this article (10.1007/s00125-020-05133-4) contains peer-reviewed but unedited supplementary material, which is available to authorised users.

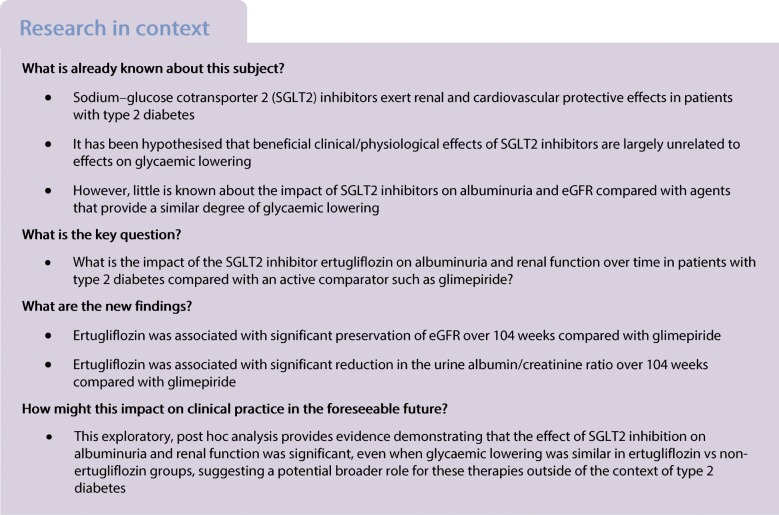



## Introduction

Beyond lowering blood glucose levels and inducing weight loss in individuals with type 2 diabetes mellitus, sodium–glucose cotransporter 2 (SGLT2) inhibitors are associated with protective renal and cardiovascular effects. These agents reduce BP by approximately 4.0 mmHg systolic and 2.0 mmHg diastolic, possibly through reducing endothelial dysfunction and arterial stiffness [1], and by inducing a modest degree of circulating volume contraction [2]. Regardless of the responsible mechanism, some SGLT2 inhibitors have been demonstrated to reduce cardiovascular events compared with placebo in renal and cardiovascular outcome trials [3–6]. However, mechanisms are still being elucidated [7–10].

At the kidney level, SGLT2 inhibitors attenuate intraglomerular hypertension and single nephron hyperfiltration by activating tubuloglomerular feedback [11, 12], a mechanism likely linked with albuminuria lowering in the setting of diabetes [13, 14]. Importantly, anti-albuminuric effects of SGLT2 inhibitors have been reported to be largely independent of other factors that influence urine albumin excretion (i.e. declines in body weight, HbA_1c_ and BP) [14]. Mechanisms related to changes in haemodynamic function, renal hypoxia and inflammation may also contribute to albuminuria-lowering effects and renal protection with SGLT2 inhibitors [7, 15, 16].

The hypothesis that SGLT2 inhibitors confer renal protection was tested in the Canagliflozin and Renal Events in Diabetes with Established Nephropathy Clinical Evaluation (CREDENCE) trial, which reported a 30% reduction in the primary composite endpoint (end-stage kidney disease, doubling of the serum creatinine level or death from renal or cardiovascular causes) [3]. The Dapagliflozin and Prevention of Adverse Outcomes in Chronic Kidney Disease (DAPA-CKD; ClinicalTrials.gov NCT03036150) and EMPA-KIDNEY studies (NCT03594110) will further assess the renal protective effects of SGLT2 inhibitors in patients with chronic kidney disease (CKD) with or without diabetes.

Ertugliflozin is a selective SGLT2 inhibitor approved for use in adults with type 2 diabetes mellitus as a glucose-lowering agent [17, 18], and is being evaluated in the ongoing cardiovascular outcome trial eValuation of ERTugliflozin effIcacy and Safety (VERTIS) CV [19]. Ertugliflozin exerts metabolic and BP effects that are comparable to other SGLT2 inhibitors [20]. Some of the longer-term renal effects of ertugliflozin, including changes in urine albumin/creatinine ratio (UACR) and renal safety, have not been reported.

Accordingly, in this post hoc, exploratory analysis, we used data from two RCTs (VERTIS MET [ClinicalTrials.gov registration no. NCT02033889] and VERTIS SU [NCT01999218]) that evaluated ertugliflozin vs glimepiride or placebo/glimepiride (non-ertugliflozin) in patients with type 2 diabetes mellitus [21, 22], as described in the electronic supplementary material (ESM) Table 1 and ESM Figs 1 and 2. These studies were combined for the purposes of this analysis as they included patients with similar background glucose-lowering medication, had the same study duration of 104 weeks and used glimepiride as the active comparator. The inclusion of studies with an active comparator was important in order to attenuate the impact of glucose lowering on exploratory outcomes. The two studies were pooled to evaluate the effects of ertugliflozin on eGFR (using the Modification of Diet in Renal Disease study equation) and albuminuria (UACR, geometric mean of change from baseline) in the overall population and by baseline UACR category over 104 weeks. The renal safety profile of ertugliflozin was assessed in the overall population.

## Methods

Data were pooled from two Phase III studies from the VERTIS programme in patients with type 2 diabetes mellitus: VERTIS MET (protocol MK-8835-007; ClinicalTrials.gov NCT02033889) and VERTIS SU (protocol MK-8835-002; ClinicalTrials.gov NCT01999218).

The studies were conducted in accordance with principles of Good Clinical Practice and were approved by the appropriate institutional review boards and regulatory agencies. Informed consent was obtained from individuals in each study. The designs for the two studies have been previously published [21–24] and are summarised in ESM Table 1. Both studies had two treatment phases and a treatment period of 104 weeks. The VERTIS MET study comprised a double-blind, placebo-controlled, 26 week treatment period (Phase A) and a double-blind, 78 week treatment extension period (Phase B; ESM Fig. 1). In Phase A, patients with an HbA_1c_ of 53–91 mmol/mol (7.0–10.5%) who were receiving metformin monotherapy were randomised to placebo, ertugliflozin 5 mg or ertugliflozin 15 mg administered once daily. In Phase B, participants who had a fasting capillary glucose ≥6.1 mmol/l and were not rescued during Phase A had the addition of blinded glimepiride (for participants randomised to placebo) or glimepiride placebo (for participants randomised to ertugliflozin). Overall, 621 participants were randomised at the start of the study (209, 207 and 205 patients in the placebo, ertugliflozin 5 mg and ertugliflozin 15 mg groups, respectively), and 581 participants entered Phase B (190, 201 and 190 participants in the glimepiride, ertugliflozin 5 mg and ertugliflozin 15 mg groups, respectively) and received ≥1 dose of study medication in Phase B [21]. The VERTIS SU study comprised a double-blind, active-controlled, 52 week treatment period (Phase A), with a double-blind, active-controlled 52 week treatment extension period (Phase B; ESM Fig. 2). In Phase A, patients with an HbA_1c_ of 53–75 mmol/mol (7.0–9.0%) who were receiving metformin monotherapy were randomised to glimepiride, ertugliflozin 5 mg or ertugliflozin 15 mg administered once daily. These treatments were continued during the Phase B extension period. Overall, 1315 participants were randomised and had ≥1 dose of study medication in the study (435, 445 and 435 patients in the glimepiride, ertugliflozin 5 mg and ertugliflozin 15 mg groups, respectively). Of these, 1037 participants entered Phase B (349, 337 and 351 patients in the glimepiride, ertugliflozin 5 mg and ertugliflozin 15 mg groups, respectively). Participants receiving glimepiride in VERTIS SU, and those receiving placebo (26 weeks) and glimepiride (78 weeks) in VERTIS MET, comprised the non-ertugliflozin control group for the purpose of all analyses reported here.

The analyses were performed on the safety population (randomised patients who took ≥1 dose of study medication) and included data after the initiation of glycaemic rescue therapy. Mean changes from baseline in eGFR and UACR over 104 weeks were evaluated using the longitudinal data analysis model. The model contained fixed effects for treatment, time, trial, treatment by time interaction and baseline covariates (HbA_1c_, systolic BP and eGFR for the eGFR analysis, and HbA_1c_, systolic BP and UACR for the UACR analysis). In this model, time was treated as a categorical variable so that no restriction was imposed on the trajectory of the means over time. The treatment difference in terms of mean change from baseline to a given time point was estimated and tested. An unstructured covariance matrix was used to model the correlation among repeated measurements. The analysis was performed on data from the overall population and patients with baseline UACR ≥3.39 mg/mmol. Due to the non-normal distribution of UACR, log transformation of UACR data was applied before analysis. Adjusted least squares means (LSMs) with 95% CIs and differences between treatments were back-transformed to the original scale. Adjusted percentage changes (derived from the exponentiation of adjusted LSMs) in UACR from baseline are presented.

The slopes for changes in eGFR per week were analysed by random coefficient models. The model included the eGFR value as a response variable with treatment group, time (in weeks), treatment by time interaction and baseline eGFR as linear covariates. The model allowed individual participant slopes to vary by random effects of intercept and time.

Adverse events (AEs) occurring up to 14 days after the final dose of study medication were included. Key endpoints included AEs related to decreased eGFR (defined by a Custom Medical Dictionary for Regulatory Activities [MedDRA; version 19.0] Query, which comprised the following preferred terms: blood creatinine increased, GFR decreased, creatinine renal clearance decreased and hypercreatininaemia); renal-related AEs (defined by the standardised MedDRA query of acute renal failure, narrow); and renal events adjudicated for causality by a blinded, external, independent committee.

Changes from baseline in metabolic, haemodynamic and volume-related biochemical variables (HbA_1c_, fasting plasma glucose, weight, systolic and diastolic BP, pulse rate, haematocrit, haemoglobin, albumin, sodium, potassium, calcium, bicarbonate, magnesium, phosphate levels and uric acid) at week 104 were assessed by treatment group in the overall population. The analysis was based on a mixed model for repeated measures with treatment, time, trial, the interaction of time by treatment and baseline covariates (HbA_1c_, eGFR and systolic BP).

## Results

A total of 1936 randomised, treated patients were included in the analyses. Of these, 644 patients received non-ertugliflozin, 652 ertugliflozin 5 mg and 640 ertugliflozin 15 mg. Baseline characteristics in the treatment groups were balanced (Table 1). Overall, mean (SD) baseline eGFR was 88.2 (18.8) ml min^−1^ (1.73 m)^−2^ and geometric mean (95% CI) of baseline UACR was 1.31 mg/mmol (1.23, 1.38). In the overall cohort, 22.5%, 23.1% and 20.1% of patients had UACR ≥3.39 mg/mmol at baseline in the non-ertugliflozin, ertugliflozin 5 mg and ertugliflozin 15 mg groups, respectively. The proportions of patients taking antihypertensive, lipid-lowering and glucose-lowering agents are shown in Table 1.

**Table 1:** Baseline demographics and disease characteristics

Variable	Non-ertugliflozin (*N* = 644)	Ertugliflozin 5 mg (*N* = 652)	Ertugliflozin 15 mg (*N* = 640)
Male, *n* (%)	322 (50.0)	324 (49.7)	284 (44.4)
Age, years	57.4 (9.0)	58.1 (9.3)	57.7 (9.7)
Race, *n* (%)			
White	461 (71.6)	463 (71.0)	444 (69.4)
Black	44 (6.8)	39 (6.0)	42 (6.6)
Asian	103 (16.0)	115 (17.6)	120 (18.8)
Ethnicity, *n* (%)			
Hispanic or Latino	131 (20.3)	125 (19.2)	121 (18.9)
Duration of type 2 diabetes mellitus, years	7.7 (5.9)	7.5 (5.8)	7.7 (5.6)
Fasting plasma glucose, mmol/l^a^	9.0 (2.0)	9.1 (2.1)	9.1 (2.1)
HbA_1c_, mmol/mol^b^	62.8 (8.0)	62.7 (7.8)	62.9 (8.1)
HbA_1c_, %^b^	7.9 (0.7)	7.9 (0.7)	7.9 (0.7)
Albumin, g/l^c^	45.0 (2.8)	44.5 (2.8)	44.7 (2.8)
Haematocrit, proportion of 1.0^d^	0.4 (0.0)	0.4 (0.0)	0.4 (0.0)
Haemoglobin, g/l^e^	138.4 (12.9)	137.9 (12.7)	137.4 (12.3)
Body weight, kg	86.1 (19.7)	87.0 (18.5)	85.5 (18.3)
BMI, kg/m^2^	31.0 (5.9)	31.4 (5.3)	31.2 (5.7)
Pulse rate, beats/min^f^	73.2 (9.3)	73.3 (9.1)	73.0 (9.6)
Systolic BP, mmHg^g^	129.7 (13.2)	130.3 (13.1)	130.6 (12.3)
Diastolic BP, mmHg^g^	77.7 (7.4)	78.0 (7.9)	77.8 (7.3)
eGFR, ml min^−1^ (1.73 m)^−2^	88.2 (19.1)	88.5 (18.3)	88.0 (19.1)
UACR, geometric mean (95% CI), mg/mmol^h^	1.26 (1.14, 1.39)	1.31 (1.18, 1.45)	1.35 (1.22, 1.50)
UACR ≥3.39 mg/mmol, *n*/*m* (%)	142/631 (22.5)	147/637 (23.1)	126/628 (20.1)
Geometric mean (95% CI)	10.30 (8.75, 12.16)	9.12 (7.85, 10.57)	9.74 (8.26, 11.48)
Uric acid, μmol/l	323.21 (88.03)	322.54 (79.71)	324.11 (80.06)
Antihypertensive therapy at screening, *n* (%)			
Diuretics	131 (20.3)	145 (22.2)	155 (24.2)
RAAS inhibitors	390 (60.6)	417 (64.0)	370 (57.8)
β-blockers	158 (24.5)	149 (22.9)	152 (23.8)
Calcium channel blockers	118 (18.3)	133 (20.4)	129 (20.2)
Antihyperglycaemic therapy at screening, *n* (%)			
Biguanides	644 (100.0)	652 (100.0)	639 (99.8)
Dipeptidyl peptidase-4 inhibitors	36 (5.6)	21 (3.2)	28 (4.4)
Sulphonylureas	113 (17.5)	119 (18.3)	105 (16.4)
Lipid-modifying agents at screening, *n* (%)	340 (52.8)	356 (54.6)	350 (54.7)

Values are mean (SD) unless otherwise stated

^a^Data from 637, 641 and 638 patients in the non-ertugliflozin, ertugliflozin 5 mg and ertugliflozin 15 mg groups, respectively

^b^Data from 642, 649 and 637 patients in the non-ertugliflozin, ertugliflozin 5 mg and ertugliflozin 15 mg groups, respectively

^c^Data from 627, 627 and 625 patients in the non-ertugliflozin, ertugliflozin 5 mg and ertugliflozin 15 mg groups, respectively

^d^Data from 612, 616 and 603 patients in the non-ertugliflozin, ertugliflozin 5 mg and ertugliflozin 15 mg groups, respectively

^e^Data from 627, 629 and 625 patients in the non-ertugliflozin, ertugliflozin 5 mg and ertugliflozin 15 mg groups, respectively

^f^Data from 636, 646 and 634 patients in the non-ertugliflozin, ertugliflozin 5 mg and ertugliflozin 15 mg groups, respectively

^g^Data from 636, 646 and 633 patients in the non-ertugliflozin, ertugliflozin 5 mg and ertugliflozin 15 mg groups, respectively

^h^Data from 631, 637 and 628 patients in the non-ertugliflozin, ertugliflozin 5 mg and ertugliflozin 15 mg groups, respectively

*m*, number of patients with data for the analysis; *n*, number of patients; RAAS, renin–angiotensin–aldosterone system

### eGFR change over time and eGFR slope in the overall cohort

**Fig. 1:**
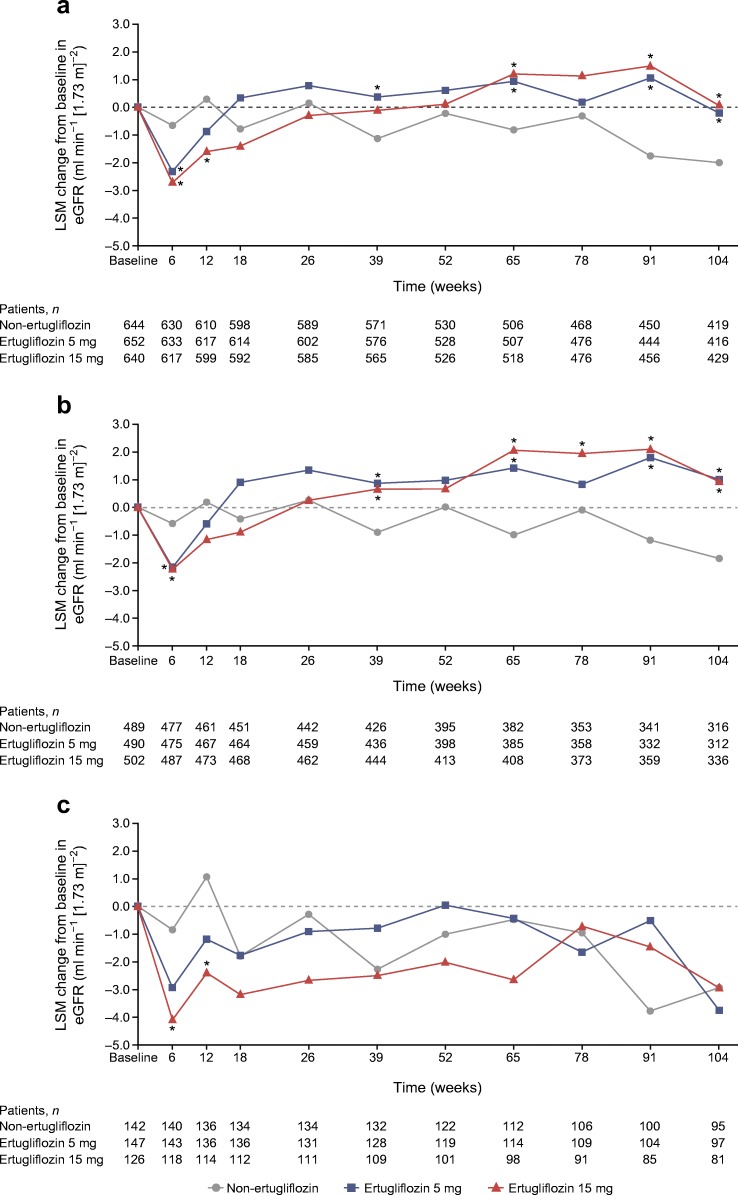
LSM change from baseline in eGFR (MDRD) over 104 weeks in (**a**) the overall cohort, (**b**) patients with a baseline UACR <3.39 mg/mmol and (**c**) patients with a baseline UACR ≥3.39 mg/mmol. *p < 0.05 for the differences between the ertugliflozin and non-ertugliflozin groups. MDRD, Modification of Diet in Renal Disease

At week 6, greater LSM reductions from baseline in eGFR were observed in the ertugliflozin groups (−2.3 and −2.7 ml min^−1^ [1.73 m]^−2^ for the ertugliflozin 5 and 15 mg groups, respectively) compared with the non-ertugliflozin group (−0.7 ml min^−1^ [1.73 m]^−2^; Fig. 1a). After week 6, eGFR in the ertugliflozin groups gradually returned to baseline. At week 104, LSM changes in eGFR from baseline were −2.0 ml min^−1^ (1.73 m)^−2^ in the non-ertugliflozin group compared with −0.2 and 0.1 ml min^−1^ (1.73 m)^−2^ for the ertugliflozin 5 and 15 mg groups, respectively.

Treatment with ertugliflozin demonstrated a different eGFR slope pattern over time compared with the non-ertugliflozin group (Table 2). In the initial 6 week period, the ertugliflozin groups had greater reductions in eGFR compared with the non-ertugliflozin group (−0.10, −0.38 and −0.45 ml min^−1^ [1.73 m]^−2^ change per week for the non-ertugliflozin, ertugliflozin 5 mg and ertugliflozin 15 mg groups, respectively); however, in weeks 6–104, the slope was positive in the ertugliflozin groups compared with a persistent negative slope in the non-ertugliflozin group (−0.01, 0.02 and 0.03 ml min^−1^ (1.73 m)^−2^ change per week for the non-ertugliflozin, ertugliflozin 5 mg and ertugliflozin 15 mg groups, respectively; *p* < 0.001).

**Table 2:** Slope analysis of eGFR (change per week, ml min^−1^ [1.73 m]^−2^)

Population	Treatment (*N/n*)	Duration, weeks	Slope (95% CI)	Slope difference (95% CI) vs non-ertugliflozin	*p* value^a^
Overall	Non-ertugliflozin (644)	0–6	−0.10 (−0.32, 0.13)		
	Ertugliflozin 5 mg (652)		−0.38 (−0.61, −0.15)	−0.29 (−0.61, 0.04)	0.08
	Ertugliflozin 15 mg (640)		−0.45 (−0.68, −0.21)	−0.35 (−0.68, −0.02)	0.04
	Non-ertugliflozin (630)	6–104	−0.01 (−0.02, 0.00)		
	Ertugliflozin 5 mg (633)		0.02 (0.01, 0.03)	0.03 (0.02, 0.04)	<0.001
	Ertugliflozin 15 mg (617)		0.03 (0.02, 0.04)	0.05 (0.04, 0.06)	<0.001
UACR <3.39 mg/mmol at baseline	Non-ertugliflozin (489)	0–6	−0.07 (−0.32, 0.18)		
	Ertugliflozin 5 mg (490)		−0.37 (−0.62, −0.12)	−0.30 (−0.65, 0.06)	0.10
	Ertugliflozin 15 mg (502)		−0.37 (−0.62, −0.12)	−0.30 (−0.65, 0.05)	0.10
	Non-ertugliflozin (477)	6–104	−0.01 (−0.02, 0.00)		
	Ertugliflozin 5 mg (475)		0.02 (0.01, 0.03)	0.03 (0.02, 0.05)	<0.001
	Ertugliflozin 15 mg (487)		0.04 (0.03, 0.05)	0.05 (0.03, 0.06)	<0.001
UACR ≥3.39 mg/mmol at baseline	Non-ertugliflozin (142)	0–6	−0.17 (−0.67, 0.33)		
	Ertugliflozin 5 mg (147)		−0.45 (−0.95, 0.04)	−0.28 (−0.98, 0.43)	0.44
	Ertugliflozin 15 mg (126)		−0.61 (−1.15, −0.07)	−0.44 (−1.18, 0.30)	0.25
	Non-ertugliflozin (140)	6–104	−0.03 (−0.05, −0.01)		
	Ertugliflozin 5 mg (143)		0.00 (−0.02, 0.02)	0.03 (0.00, 0.06)	0.05
	Ertugliflozin 15 mg (118)		0.01 (−0.01, 0.03)	0.04 (0.01, 0.07)	0.01

^a^Testing the slope difference between the ertugliflozin and non-ertugliflozin groups

### eGFR change over time and eGFR slope by baseline UACR

In patients without albuminuria (UACR <3.39 mg/mmol) at baseline, LSM changes in eGFR from baseline to week 6 were −0.6, −2.2 and −2.2 ml min^−1^ (1.73 m)^−2^ for the non-ertugliflozin, ertugliflozin 5 mg and ertugliflozin 15 mg groups, respectively (Fig. 1b). In these patients, after week 6, eGFR increased over time in the ertugliflozin groups and decreased in the non-ertugliflozin group. eGFR increased to above baseline in the ertugliflozin groups by week 104. At week 104, LSM changes from baseline in eGFR were −1.9, 1.0 and 1.0 ml min^−1^ (1.73 m)^−2^ for the non-ertugliflozin, ertugliflozin 5 mg and ertugliflozin 15 mg groups, respectively.

In patients with albuminuria (UACR ≥3.39 mg/mmol) at baseline, results at week 6 were similar to those in the overall cohort, with slightly larger decreases in eGFR noted in patients in the ertugliflozin groups (Fig. 1c). In patients with albuminuria at baseline, LSM changes in eGFR from baseline to week 6 were −0.8, −3.0 and −4.1 ml min^−1^ (1.73 m)^−2^ for the non-ertugliflozin, ertugliflozin 5 mg and ertugliflozin 15 mg groups, respectively. Over the remaining 98 weeks, eGFR tended to remain at similar levels in the ertugliflozin groups, while it tended to decrease in the non-ertugliflozin group. At week 104, LSM changes from baseline in eGFR were −2.9, −3.8 and −2.9 ml min^−1^ (1.73 m)^−2^ for the non-ertugliflozin, ertugliflozin 5 mg and ertugliflozin 15 mg groups, respectively.

In patients without albuminuria at baseline, eGFR slope values were similar to the overall cohort, with significant differences in eGFR slope in weeks 6 to 104 between the ertugliflozin groups and the non-ertugliflozin group (Table 2). In patients with albuminuria at baseline, there was a significant decline in eGFR from week 6 to week 104 in the non-ertugliflozin group, while the eGFR remained stable in the ertugliflozin groups (Table 2). The between-group difference (95% CI) in slope from week 6 to week 104 was 0.04 (0.01, 0.07) ml min^−1^ (1.73 m)^−2^ per week, which was significant between the non-ertugliflozin and ertugliflozin 15 mg treatment arms (*p* = 0.01; Table 2). Compared with the non-ertugliflozin group slope, change in slope with ertugliflozin treatment was approximately equivalent for both albuminuria subgroups.

### UACR change over time

**Fig. 2:**
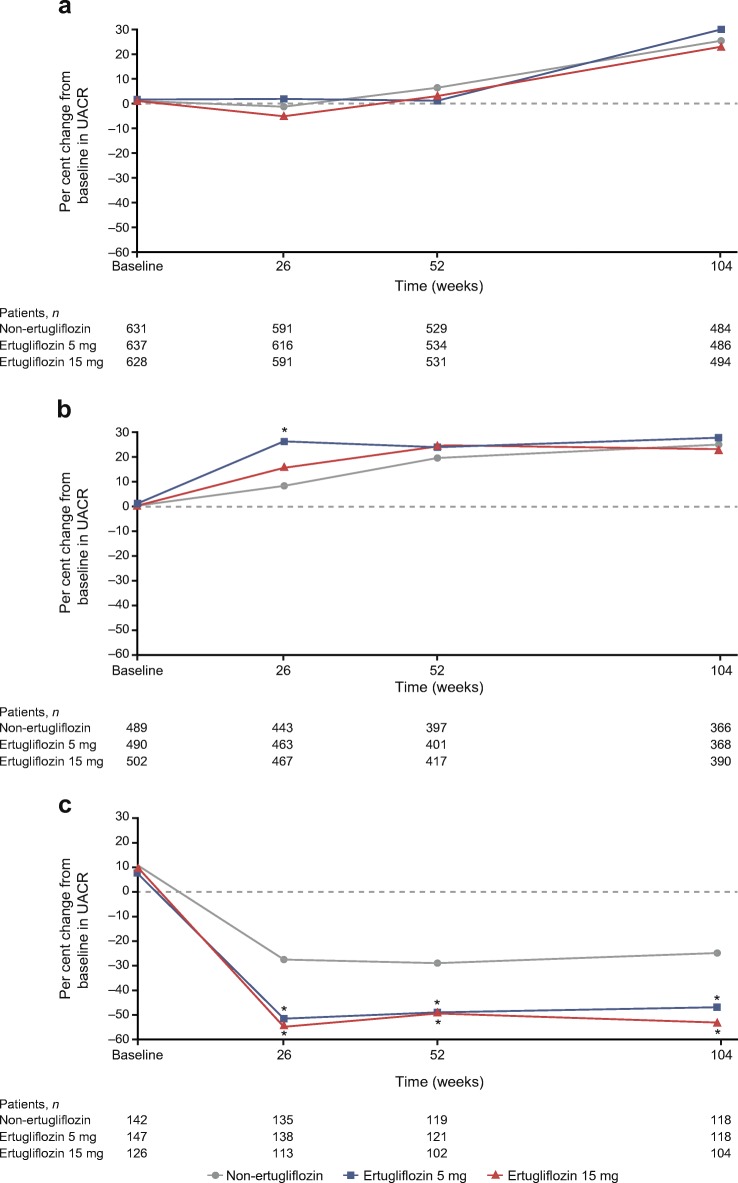
Per cent change from baseline in UACR in pooled 104 week studies in (**a**) the overall cohort, (**b**) patients with a baseline UACR <3.39 mg/mmol and (**c**) patients with a baseline UACR ≥3.39 mg/mmol. *p < 0.05 for the difference between the ertugliflozin and non-ertugliflozin groups

At week 104, changes from baseline in UACR (95% CI) in the ertugliflozin 5 and 15 mg groups compared with the non-ertugliflozin group in the overall cohort were 3.6% (−6.9, 15.2; *p* = 0.52) and −1.6% (−11.4, 9.2; *p* = 0.76), respectively (Fig. 2a).

In patients without albuminuria at baseline (*n* = 1482), although a significant difference was observed in the change from baseline in UACR (95% CI) at week 26 in the ertugliflozin 5 mg group compared with the non-ertugliflozin group (16.6% [6.5, 27.6]; *p* < 0.001), this difference had attenuated by week 104 as no further meaningful differences between the three groups were observed (Fig. 2b).

In patients with albuminuria at baseline (*n* = 415; Fig. 2c), the ertugliflozin groups had greater UACR reductions at week 26 compared with the non-ertugliflozin group. At week 104, change from baseline in UACR (95% CI) compared with the non-ertugliflozin group was −29.5% (−44.8, −9.8; *p* < 0.01) for ertugliflozin 5 mg and −37.6% (−51.8, −19.2; *p* < 0.001) for ertugliflozin 15 mg.

The interactions between reductions from baseline in HbA_1c_ (>6, >3 to ≤6 or ≤3 mmol/mol [>0.5, >0.3 to ≤0.5 or ≤0.3%]) and changes from baseline in UACR and eGFR were assessed (ESM Tables 2–3). At week 104, non-ertugliflozin-adjusted reductions from baseline in UACR with ertugliflozin were observed in the HbA_1c_-lowering category of >3 to ≤6 mmol/mol (>0.3 to ≤0.5%; up to 3.5 mg/mmol with ertugliflozin 5 mg; *p* < 0.05), but not in the reduction category >6 mmol/mol (>0.5%). Non-ertugliflozin-adjusted reduction from baseline in UACR with ertugliflozin 15 mg of 4.5 mg/mmol was observed in the HbA1c-lowering category of ≤3 mmol/mol (≤0.3%); however, this result was not statistically significant (*p* = 0.11). At week 104, non-ertugliflozin-adjusted changes from baseline in eGFR with ertugliflozin were observed in HbA_1c_-lowering categories of >6 (>0.5%; up to 2.6 ml min^−1^ [1.73 m]^−2^ with ertugliflozin 15 mg; *p* < 0.05) and >3 to ≤6 mmol/mol (>0.3 to ≤0.5%; up to 7.1 ml min^−1^ [1.73 m]^−2^ with ertugliflozin 15 mg; *p* = 0.01), but not in the reduction category ≤3 mmol/mol (≤0.3%).

### Changes in metabolic and haemodynamic variables in the pooled population

Ertugliflozin was associated with significant decreases from baseline in fasting plasma glucose, HbA_1c_, systolic BP and body weight, and statistically significant increases in haemoglobin and haematocrit, at week 104 (Table 3). Compared with treatment with non-ertugliflozin, ertugliflozin was also associated with a significant increase in serum albumin at week 104 (Table 3). Consistent with the results of the individual studies, reductions in HbA_1c_ from baseline at week 104 were similar between ertugliflozin and non-ertugliflozin.

**Table 3:** Summary of metabolic, haemodynamic and volume-related biochemical changes from baseline at week 104

Variable	Treatment group	*n*	LSM (95% CI)	Difference vs non-ertugliflozin	
				LSM (95% CI)	*p* value^a^
HbA_1c_, mmol/mol	Non-ertugliflozin	417	−6.84 (−7.65, −6.03)		
	Ertugliflozin 5 mg	420	−6.84 (−7.64, −6.03)	0.0 (−1.14, 1.14)	>0.99
	Ertugliflozin 15 mg	428	−7.74 (−8.54, −6.94)	−0.90 (−2.04, −0.23)	0.12
HbA_1c_ (%)	Non-ertugliflozin	417	−0.63 (−0.70, −0.55)		
	Ertugliflozin 5 mg	420	−0.63 (−0.70, −0.55)	0.0 (−0.10, 0.10)	>0.99
	Ertugliflozin 15 mg	428	−0.71 (−0.78, −0.64)	−0.08 (−0.19, −0.02)	0.12
Fasting plasma glucose, mmol/l	Non-ertugliflozin	409	−0.73 (−0.89, −0.58)		
	Ertugliflozin 5 mg	410	−1.07 (−1.22, −0.92)	−0.34 (−0.55, −0.12)	<0.01
	Ertugliflozin 15 mg	428	−1.33 (−1.48, −1.18)	−0.60 (−0.81, −0.38)	<0.001
Weight, kg	Non-ertugliflozin	421	0.65 (0.35, 0.95)		
	Ertugliflozin 5 mg	419	−3.24 (−3.54, −2.94)	−3.89 (−4.31, −3.47)	<0.001
	Ertugliflozin 15 mg	431	−3.48 (−3.77, −3.18)	−4.13 (−4.55, −3.71)	<0.001
Systolic BP, mmHg	Non-ertugliflozin	413	1.70 (0.64, 2.75)		
	Ertugliflozin 5 mg	415	−2.81 (−3.86, −1.75)	−4.50 (−6.00, −3.02)	<0.001
	Ertugliflozin 15 mg	426	−1.82 (−2.86, −0.78)	−3.52 (−5.00, −2.04)	<0.001
Diastolic BP, mmHg	Non-ertugliflozin	413	0.24 (−0.43, 0.90)		
	Ertugliflozin 5 mg	415	−1.61 (−2.28, −0.95)	−1.85 (−2.79, −0.91)	<0.001
	Ertugliflozin 15 mg	426	−0.49 (−1.15, 0.16)	−0.73 (−1.67, 0.20)	0.12
Pulse rate, beats/min	Non-ertugliflozin	413	0.25 (−0.46, 0.95)		
	Ertugliflozin 5 mg	414	−0.41 (−1.11, 0.30)	−0.66 (−1.65, 0.34)	0.20
	Ertugliflozin 15 mg	426	0.35 (−0.35, 1.04)	0.10 (−0.89, 1.09)	0.85
Haematocrit, proportion of 1.0	Non-ertugliflozin	393	0.00 (−0.00, 0.00)		
	Ertugliflozin 5 mg	403	0.02 (0.02, 0.02)	0.02 (0.02, 0.02)	<0.001
	Ertugliflozin 15 mg	410	0.02 (0.02, 0.03)	0.02 (0.02, 0.03)	<0.001
Haemoglobin, g/l	Non-ertugliflozin	407	−0.14 (−0.95, 0.67)		
	Ertugliflozin 5 mg	412	6.22 (5.41, 7.03)	6.36 (5.22, 7.51)	<0.001
	Ertugliflozin 5 mg	424	7.13 (6.33, 7.92)	7.26 (6.13, 8.40)	<0.001
Albumin, g/l	Non-ertugliflozin	407	−0.47 (−0.67, −0.27)		
	Ertugliflozin 5 mg	402	0.00 (−0.21, 0.20)	0.47 (0.18, 0.76)	<0.01
	Ertugliflozin 15 mg	417	0.03 (−0.17, 0.23)	0.50 (0.20, 0.79)	<0.001
Uric acid, μmol/l	Non-ertugliflozin	406	15.50 (9.95, 21.06)		
	Ertugliflozin 5 mg	402	−28.76 (−34.33, −23.19)	−44.27 (−52.13, −36.40)	<0.001
	Ertugliflozin 15 mg	417	−34.57 (−40.04, −29.10)	−50.07 (−57.87, −42.28)	<0.001

^a^Testing the difference between the ertugliflozin and non-ertugliflozin groups

### Safety profile

The incidence of AEs related to decreased eGFR was low across groups but was higher in the ertugliflozin groups compared with the non-ertugliflozin group (non-ertugliflozin, 0.5%; ertugliflozin 5 mg, 0.8%; and ertugliflozin 15 mg, 1.7%). One AE was serious (eGFR decreased in the ertugliflozin 15 mg group); few patients discontinued treatment due to these AEs (non-ertugliflozin, 0.2%; ertugliflozin 5 mg, 0.0%; and ertugliflozin 15 mg, 0.5%).

The incidence of renal-related AEs at 104 weeks was low and not notably different across groups (non-ertugliflozin, 0.3%; ertugliflozin 5 mg, 0.3%; and ertugliflozin 15 mg, 0.6%; ESM Table 4). One AE was serious (acute kidney injury in the ertugliflozin 15 mg group). A small number of patients discontinued treatment due to renal-related AEs (non-ertugliflozin, 0.0%; ertugliflozin 5 mg, 0.2%; and ertugliflozin 15 mg, 0.3%). Few patients had events that were adjudicated as causally related to study medication (‘possible’ or ‘very likely’): there were none in the non-ertugliflozin or ertugliflozin 5 mg groups and two in the ertugliflozin 15 mg group.

At week 104, there were no meaningful changes from baseline in serum sodium, potassium, calcium or bicarbonate with ertugliflozin relative to non-ertugliflozin. However, there were small increases in serum magnesium and phosphate, while there were small decreases in uric acid (ESM Table 5).

## Discussion

In this exploratory analysis, ertugliflozin was associated with an initial, early dip in eGFR, followed by a return in eGFR back to or above baseline levels by 104 weeks. By contrast, eGFR decreased over time in the non-ertugliflozin group. These findings were observed even though changes in HbA_1c_ were comparable in ertugliflozin vs non-ertugliflozin treatment groups. In addition, among patients with albuminuria at baseline, the ertugliflozin groups had greater reductions in UACR at all measured time points up to week 104 even though changes in HbA_1c_ were similar vs non-ertugliflozin therapy. The longer-term renal safety profile of ertugliflozin was generally consistent with observations made with other SGLT2 inhibitors [4, 13].

Experimental models of diabetes and mechanistic studies in humans have shown that SGLT2 inhibition reduces renal hyperfiltration through activation of tubuloglomerular feedback, leading to afferent arteriolar vasoconstriction and a decline in intraglomerular pressure [10, 25, 26]. Importantly, hyperfiltration is associated with CKD initiation and progression, and a variety of physiological factors that promote CKD progression [27–29]. Reduced glomerular pressure by SGLT2 inhibition is also mediated by increases in tubular pressure that reduce net filtration pressure and glomerular hypertension [12, 30]. In patients with type 2 diabetes mellitus, albuminuria decreases by 7–41% after 12 weeks in response to SGLT2 inhibition with empagliflozin, and stays low even after 3 years of treatment in clinical studies [13, 14]. This is similar to experimental models and is a consequence of changes in renal physiology [31].

Similar to observations in experimental studies demonstrating SGLT2 inhibition effects on intraglomerular hypertension and hyperfiltration, these agents reduce inulin-based measures of hyperfiltration and hyperperfusion in human mechanistic studies [11]. In larger clinical studies, SGLT2 inhibitors acutely induce a dip in eGFR [32, 33], which happens even after a single dose of drug [34] and tends to return towards baseline over time. Importantly, after a 2–4 week washout period, eGFR tends to return to baseline even after prolonged periods of therapy [35, 36]. This pattern of renal function change is thought to reflect known haemodynamic effects of SGLT2 inhibitors in the renal microcirculation. In a similar fashion to these previous observations with other SGLT2 inhibitor agents, ertugliflozin induced an eGFR dip during weeks 0–6 in the current exploratory analysis. Based on known mechanisms, changes in renal function are mainly attributed to sodium-related pathways [37], rather than renal glucose handling or glycaemic lowering [7, 8, 15, 38]. Similar to effects on BP lowering [39], the characteristic early dip in eGFR occurs in patients with normal kidney function and in those with CKD stages 2–4 [35, 40].

Interestingly, in the current analysis, after the initial dip, eGFR tended to increase back towards or above baseline over time. These findings are consistent with the pharmacodynamic effects of ertugliflozin. While the mechanisms responsible for the eGFR dip have relatively accepted explanations [16, 41, 42], the mechanism for the rise in eGFR back to and above baseline is incompletely understood but may be related to compensatory upregulation of sodium–glucose cotransporter 1 [43]. This results in increased sodium–glucose cotransport and afferent arteriolar redilatation, resulting in increases in renal perfusion and GFR. Aside from hypothetical pathways leading to the rise in eGFR over time, similar increases in eGFR towards baseline levels have been reported in the Canagliflozin Cardiovascular Assessment Study (CANVAS) and EMPA-REG OUTCOME studies [13, 44]. The clinical relevance of this pattern of renal function change over time is not yet understood but appears in part to reflect preservation of renal function. Beyond these possible haemodynamic effects in the kidney, ertugliflozin was associated with increases from baseline in serum albumin, haemoglobin and haematocrit—changes that have been most closely associated with haemoconcentration in the systemic circulation on the basis of natriuresis and osmotic diuresis. While these volume-related effects have been linked with cardiovascular protection, especially against heart failure hospitalisation [10, 45], their clinical relevance is not currently known. Interestingly, despite the natriuresis and volume-related effects attributed to SGLT2 inhibitors, as with previous work, ertugliflozin did not impact plasma electrolyte levels in a meaningful way, aside from small changes in magnesium and phosphate that were unlikely to be clinically significant. As expected based on previous work, uric acid decreased—an effect that has been attributed to glucosuria-related uricosuria, and associated with cardiovascular benefits in exploratory mediation analyses from the EMPA-REG OUTCOME study [45, 46].

Ertugliflozin is a highly selective SGLT2 inhibitor approved for use as a glucose-lowering therapy in patients with type 2 diabetes mellitus. Beyond changes in eGFR in the current analysis, ertugliflozin also reduced UACR in patients with elevated albuminuria at baseline. In previous work, empagliflozin reduced UACR in patients with type 2 diabetes mellitus with either baseline micro- or macroalbuminuria [13]. These changes were largely independent of changes in body weight, BP or HbA_1c_. In other work, Heerspink et al. [47, 48] demonstrated that dapagliflozin and canagliflozin similarly reduced UACR—effects that were also largely independent of changes in these variables. The current analysis in a large study cohort using an active glucose-lowering comparator minimised differences in HbA_1c_ over time, supporting observations from placebo-controlled trials and suggesting that favourable effects on eGFR and albuminuria are not due to differences in glycaemic control, which is a more distinct feature of this analysis. Moreover, in this analysis we examined both acute and chronic eGFR changes in this cohort. One caveat is that, compared with non-ertugliflozin-treated patients, preservation of eGFR was only apparent in ertugliflozin-treated patients in the >6 (>0.5%) and >3 to ≤6 mmol/mol (>0.3 to ≤0.5%) HbA_1c_ reduction categories, but not in the treatment groups with ≤3 mmol/mol (≤0.3%) HbA_1c_ reduction, at week 104. Non-ertugliflozin-adjusted reductions from baseline in UACR in ertugliflozin-treated patients were also observed in the >3 to ≤6 mmol/mol (>0.3 to ≤0.5%) HbA_1c_ reduction category compared with groups in the other HbA_1c_-lowering categories. Therefore, an interaction between the degree of HbA_1c_ lowering and kidney protection cannot be ruled out. Based on existing data, it may be speculated that, rather than being based on alterations in body weight, BP or HbA_1c_, reductions in albuminuria and eGFR preservation are based primarily on reduced glomerular hypertension or improvements in glomerular membrane barrier function [49]—a hypothesis that is being tested in dedicated kidney outcome studies [50].

In the context of previous work demonstrating that changes in UACR are largely independent of other clinical factors that reduce albuminuria [14], our analysis emphasises that, even when compared with a sulfonylurea with comparable glucose-lowering efficacy, ertugliflozin reduced UACR. It is therefore important to define possible albuminuria-lowering and renal protective effects of SGLT2 inhibitors in patients with non-diabetic kidney disease, where hyperglycaemia is not implicated in kidney disease progression. This critical question is being examined in mechanistic studies, such as the Effects of Dapagliflozin in Non-diabetic Patients With Proteinuria (DIAMOND) study (ClinicalTrials.gov registration no. NCT03190694), and in long-term renal outcome studies (DAPA-CKD [NCT03036150] and EMPA-KIDNEY [NCT03594110]), which are including patients with non-diabetic kidney disease. These last two trials will test the hypothesis that renal protective effects of SGLT2 inhibition are independent of changes in ambient glucose levels. Similarly, for ertugliflozin specifically, the VERTIS CV trial (ClinicalTrials.gov registration no. NCT01986881), which is being performed in over 8000 patients with established cardiovascular disease to assess cardiovascular safety, may elucidate possible renal protective effects with this agent since kidney outcomes are important secondary endpoints [19].

This analysis has some limitations. First, it was post hoc and exploratory in nature. Therefore, randomisation was not stratified by the baseline albuminuria status in either of the two studies. Nevertheless, the study groups were balanced in the mean baseline UACR levels and the proportions of patients with baseline elevations of albuminuria. Second, there was no type 1 error control for multiple testing in the analyses. The nominal *p* values were reported from the analysis model. Third, analysis time points for UACR and eGFR were the common measurements collected from both studies through week 104. There was no adjustment for different study durations of Phase A and Phase B from these two studies. We also recognise that the impact of SGLT2 inhibition on UACR has been reported elsewhere. Nevertheless, given that SGLT2 inhibitors have demonstrated reduced renal risk across multiple studies of different drugs, it seems likely that renal protective effects are ubiquitous across different members of the drug class.

In conclusion, transient and modest reductions in eGFR observed in the ertugliflozin groups at week 6 gradually returned to baseline by week 104. By contrast, eGFR declined from baseline through week 104 in the non-ertugliflozin group. The results are consistent with the pharmacodynamic effect seen with SGLT2 inhibitors and are suggestive of preservation of renal function with ertugliflozin. In patients with baseline albuminuria, ertugliflozin demonstrated a reduction in albuminuria over 104 weeks.

## Electronic supplementary material

ESM 1 (PDF 350 kb)

## Data Availability

The data sharing policy of Merck Sharp & Dohme Corp., a subsidiary of Merck & Co., Inc., Kenilworth, NJ, USA, including restrictions, is available at http://engagezone.msd.com/ds_documentation.php. Requests for access to the clinical study data can be submitted through the EngageZone site or via email to dataaccess@merck.com.

## Abbreviations

AE: Adverse event
CKD: Chronic kidney disease
DAPA-CKD: Dapagliflozin and Prevention of Adverse Outcomes in Chronic Kidney Disease
LSM: Least squares mean
MedDRA: Medical Dictionary for Regulatory Activities
SGLT2: Sodium–glucose cotransporter 2
UACR: Urine albumin/creatinine ratio
VERTIS: eValuation of ERTugliflozin effIcacy and Safety
