# Supplementary material for: Effects of ertugliflozin on renal function over 104 weeks of treatment: a post hoc analysis of two randomised controlled trials
Source: Diabetologia. 2020 Mar 31;63(6):1128–40. doi: 10.1007/s00125-020-05133-4 (PMC7228910; doi:10.1007/s00125-020-05133-4)
Supplement: Supplementary file 1 — (PDF 350 kb) [file 125_2020_5133_MOESM1_ESM.pdf]

Electronic Supplementary Materials

Tables

ESM Table 1 Phase III VERTIS clinical studies included in the pooled analysis

	VERTIS MET	VERTIS SU
ClincalTrials.gov identifier	NCT02033889	NCT01999218
N (randomised)	621	1316[a]
N at baseline (non-ertugliflozin/ertugliflozin 5mg /ertugliflozin 15 mg)	209/207/205	435/445/435
N at start of Phase B (non-ertugliflozin/ertugliflozin 5mg/ ertugliflozin 15 mg)[b]	190/201/190	349/337/351
Inclusion criteria		
Background antihyperglycaemic therapy	Metformin (≥1500 mg/day for ≥8 weeks)	
HbA_{1c} (inclusive), mmol/mol (%)	53–91 (7.0–10.5)	53–75 (7.0–9.0)
eGFR, mL min^{-1} 1.73 m^{-2}	≥55	
Comparator	Placebo/glimepiride[c]<br>Patients received placebo for the first 26 weeks of the study and then blinded glimepiride for the remaining 78 weeks	Glimepiride[a]

[a]One patient randomised to ertugliflozin 15 mg did not take study medication and is not included in the pooled analysis. [b]Phase B started at week 26 and week 52 in the VERTIS MET and VERTIS SU studies, respectively. [c]Glimepiride was initiated at 1 mg/day and up-titrated to a maximum of 6 or 8 mg/day according to the local label or maximum tolerated dose

ESM Table 2 Change in UACR by categories of changes from baseline in HbA_{1c} at 26, 52 and 104 weeks

Time point	Category of reduction in HbA_{1c} (mmol/mol [%])	Treatment group	*n*	Mean change in UACR from baseline (mg/mmol [SD])	Difference vs non-ertugliflozin	
					LSM (mg/mmol [95% CI])	*p* value[a]
Week 26	>6 (>0.5)	Non-ertugliflozin	337	−1.4 (30.7)	–	–
		Ertugliflozin 5 mg	315	−0.2 (11.5)	1.2 (−1.8, 4.2)	0.45
		Ertugliflozin 15 mg	332	−1.2 (9.4)	0.2 (−2.8, 3.2)	0.91
	>3 to ≤6 (>0.3 to ≤0.5)	Non-ertugliflozin	72	0.7 (10.5)	–	–
		Ertugliflozin 5 mg	95	0.3 (4.5)	−0.4 (−2.7, 1.9)	0.71
		Ertugliflozin 15 mg	89	−0.9 (6.9)	−1.7 (−4.0, 0.6)	0.15
	≤3 (≤0.3)	Non-ertugliflozin	152	1.3 (7.0)	–	–
		Ertugliflozin 5 mg	174	0.3 (11.5)	−1.0 (−2.8, 0.8)	0.27
		Ertugliflozin 15 mg	147	−0.1 (3.8)	−1.4 (−3.3, 0.5)	0.14
Week 52	>6 (>0.5)	Non-ertugliflozin	328	−1.5 (29.5)	–	–
		Ertugliflozin 5 mg	272	−1.3 (8.4)	0.2 (−2.8, 3.3)	0.88

Time point	Category of reduction in HbA_{1c} (mmol/mol [%])	Treatment group	*n*	Mean change in UACR from baseline (mg/mmol [SD])	Difference vs non-ertugliflozin	
					LSM (mg/mmol [95% CI])	*p* value[a]
		Ertugliflozin 15 mg	315	−0.6 (9.7)	0.9 (−2.0, 3.9)	0.53
	>3 to ≤6 (>0.3 to ≤0.5)	Non-ertugliflozin	57	0.7 (8.9)	–	–
		Ertugliflozin 5 mg	90	−0.1 (4.1)	−0.8 (−3.2, 1.5)	0.49
		Ertugliflozin 15 mg	72	1.1 (8.2)	0.4 (−2.1, 2.8)	0.76
	≤3 (≤0.3)	Non-ertugliflozin	121	0.8 (12.4)	–	–
		Ertugliflozin 5 mg	144	1.9 (16.5)	1.1 (−1.9, 4.1)	0.47
		Ertugliflozin 15 mg	121	0.1 (4.3)	−0.6 (−3.8, 2.5)	0.69
Week 104	>6 (>0.5)	Non-ertugliflozin	221	−0.9 (36.5)	–	–
		Ertugliflozin 5 mg	211	−1.1 (11.9)	−0.2 (−4.6, 4.2)	0.94
		Ertugliflozin 15 mg	224	−0.4 (11.8)	0.5 (−3.8, 4.9)	0.81
	>3 to ≤6 (>0.3 to ≤0.5)	Non-ertugliflozin	51	1.8 (11.2)	–	–
		Ertugliflozin 5 mg	60	−1.7 (10.5)	−3.5 (−7.0, −0.0)	<0.05

Time point	Category of reduction in HbA_{1c} (mmol/mol [%])	Treatment group	*n*	Mean change in UACR from baseline (mg/mmol [SD])	Difference vs non-ertugliflozin	
					LSM (mg/mmol [95% CI])	*p* value[a]
		Ertugliflozin 15 mg	62	−0.8 (5.3)	−2.6 (−6.1, 0.8)	0.13
	≤3 (≤0.3)	Non-ertugliflozin	132	4.1 (36.0)	–	–
		Ertugliflozin 5 mg	135	−0.1 (7.6)	−4.2 (−9.5, 1.2)	0.13
		Ertugliflozin 15 mg	122	−0.4 (11.1)	−4.5 (−10.0, 1.0)	0.11

ESM Table 3 Change in eGFR by categories of changes from baseline in HbA_{1c} at 26, 52 and 104 weeks

Time point	Category of reduction in HbA_{1c} (mmol/mol)	Treatment group	*n*	Mean change in eGFR from baseline (mL min^{-1} 1.73 m^{-2} [SD])	Difference vs non-ertugliflozin	
					LSM (mg/mmol [95% CI])	*p* value[a]
Week 26	>6 (>0.5)	Non-ertugliflozin	346	−0.2 (10.8)	–	–
		Ertugliflozin 5 mg	318	2.1 (13.5)	2.4 (0.4, 4.3)	0.02
		Ertugliflozin 15 mg	341	1.0 (13.7)	1.3 (−0.6, 3.2)	0.20
	>3 to ≤6 (>0.3 to ≤0.5)	Non-ertugliflozin	74	0.6 (9.5)	–	–
		Ertugliflozin 5 mg	96	0.3 (13.6)	−0.3 (−4.2, 3.6)	0.87
		Ertugliflozin 15 mg	90	0.0 (13.8)	−0.5 (−4.5, 3.4)	0.79
	≤3 (≤0.3)	Non-ertugliflozin	160	0.8 (11.5)	–	–
		Ertugliflozin 5 mg	181	−0.9 (12.0)	−1.7 (−4.3, 0.8)	0.18
		Ertugliflozin 15 mg	150	−3.2 (12.2)	−4.0 (−6.7, −1.4)	<0.01
Week 52	>6 (>0.5)	Non-ertugliflozin	335	−0.0 (13.0)	–	–
		Ertugliflozin 5 mg	277	1.7 (12.5)	1.7 (−0.4, 3.8)	0.11

Time point	Category of reduction in HbA_{1c} (mmol/mol)	Treatment group	*n*	Mean change in eGFR from baseline (mL min^{-1} 1.73 m^{-2} [SD])	Difference vs non-ertugliflozin	
					LSM (mg/mmol [95% CI])	*p* value[a]
		Ertugliflozin 15 mg	325	1.2 (13.6)	1.3 (−0.7, 3.2)	0.21
	>3 to ≤6 (>0.3 to ≤0.5)	Non-ertugliflozin	60	0.9 (11.2)	–	–
		Ertugliflozin 5 mg	93	0.8 (12.7)	−0.1 (−4.0, 3.8)	0.96
		Ertugliflozin 15 mg	71	−1.0 (11.8)	−1.9 (−6.1, 2.3)	0.37
	≤3 (≤0.3)	Non-ertugliflozin	130	−0.2 (12.9)	–	–
		Ertugliflozin 5 mg	150	−0.7 (12.7)	−0.5 (−3.5, 2.5)	0.73
		Ertugliflozin 15 mg	124	−2.3 (12.5)	−2.2 (−5.3, 1.0)	0.17
Week 104	>6 (>0.5)	Non-ertugliflozin	225	−2.1 (13.2)	–	–
		Ertugliflozin 5 mg	212	0.5 (13.2)	2.6 (−0.0, 5.2)	0.05
		Ertugliflozin 15 mg	230	0.5 (15.4)	2.6 (0.0, 5.2)	<0.05
	>3 to ≤6 (>0.3 to ≤0.5)	Non-ertugliflozin	52	−5.3 (15.2)	–	–
		Ertugliflozin 5 mg	61	−0.2 (14.2)	5.1 (−0.5, 10.7)	0.07

Time point	Category of reduction in HbA_{1c} (mmol/mol)	Treatment group	*n*	Mean change in eGFR from baseline (mL min^{-1} 1.73 m^{-2} [SD])	Difference vs non-ertugliflozin	
					LSM (mg/mmol [95% CI])	*p* value[a]
		Ertugliflozin 15 mg	63	1.9 (15.7)	7.1 (1.6, 12.7)	0.01
	≤3 (≤0.3)	Non-ertugliflozin	136	−0.6 (12.9)	–	–
		Ertugliflozin 5 mg	139	0.1 (13.2)	0.7 (−2.4, 3.8)	0.66
		Ertugliflozin 15 mg	128	−1.8 (13.3)	−1.2 (−4.4, 2.0)	0.46

ESM Table 4 Incidence of renal-related AEs by system organ class and preferred term

**Patients with ≥1 renal-related AE, *n* (%)**	**Non-ertugliflozin (*N*=644)**	**Ertugliflozin 5 mg (*N*=652)**	**Ertugliflozin 15 mg (*N*=640)**
Renal and urinary disorders	2 (0.3)	2 (0.3)	4 (0.6)
Acute kidney injury	0 (0.0)	0 (0.0)	2 (0.3)
Renal failure	0 (0.0)	0 (0.0)	1 (0.2)
Renal impairment	2 (0.3)	2 (0.3)	1 (0.2)

ESM Table 5 Summary of electrolyte changes from baseline at week 104

		Non-ertugliflozin (*N*=644)	Ertugliflozin 5 mg (*N*=652)	Ertugliflozin 15 mg (*N*=640)
Sodium, mmol/l				
	Baseline (SD)	*n* = 627<br>141.30 (2.55)	*n* = 628<br>141.11 (2.49)	*n* = 625<br>141.18 (2.57)
	Change from baseline	*n* = 406<br>–0.87 (2.72)	*n* = 401<br>–0.34 (2.50)	*n* = 417<br>–0.33 (2.60)
Potassium, mmol/l				
	Baseline (SD)	*n* = 627<br>4.58 (0.44)	*n* = 626<br>4.54 (0.40)	*n* = 621<br>4.54 (0.38)
	Change from baseline	*n* = 404<br>–0.05 (0.49)	*n* = 399<br>–0.06 (0.37)	*n* = 413<br>–0.06 (0.39)
Calcium, mmol/l				
	Baseline (SD)	*n* = 626<br>2.4 (0.1)	*n* = 625<br>2.4 (0.1)	*n* = 621<br>2.4 (0.1)
	Change from baseline	*n* = 406<br>−0.02 (0.15)	*n* = 400<br>−0.01 (0.11)	*n* = 413<br>−0.01 (0.11)
Bicarbonate, mmol/l				
	Baseline (SD)	*n* = 607<br>23.6 (2.3)	*n* = 612<br>23.7 (2.5)	*n* = 602<br>23.6 (2.5)
	Change from baseline	*n* = 389<br>0.43 (2.61)	*n* = 388<br>0.16 (2.71)	*n* = 399<br>−0.18 (2.70)
Magnesium, mEq/l				
	Baseline (SD)	*n* = 627<br>0.8 (0.1)	*n* = 627<br>0.8 (0.1)	*n* = 621<br>0.8 (0.1)
	Change from baseline	*n* = 406<br>−0.00 (0.07)	*n* = 401<br>0.04 (0.08)	*n* = 413<br>0.06 (0.07)
Phosphate, mmol/l				
	Baseline (SD)	*n* = 626	*n* = 627	*n* = 625

		1.2 (0.2)	1.2 (0.2)	1.2 (0.2)
	Change from baseline	*n* = 406<br>0.00 (0.16)	*n* = 401<br>0.02 (0.15)	*n* = 417<br>0.05 (0.16)

Data are presented as mean (SD)

Figures

ESM Fig. 1 VERTIS MET study design

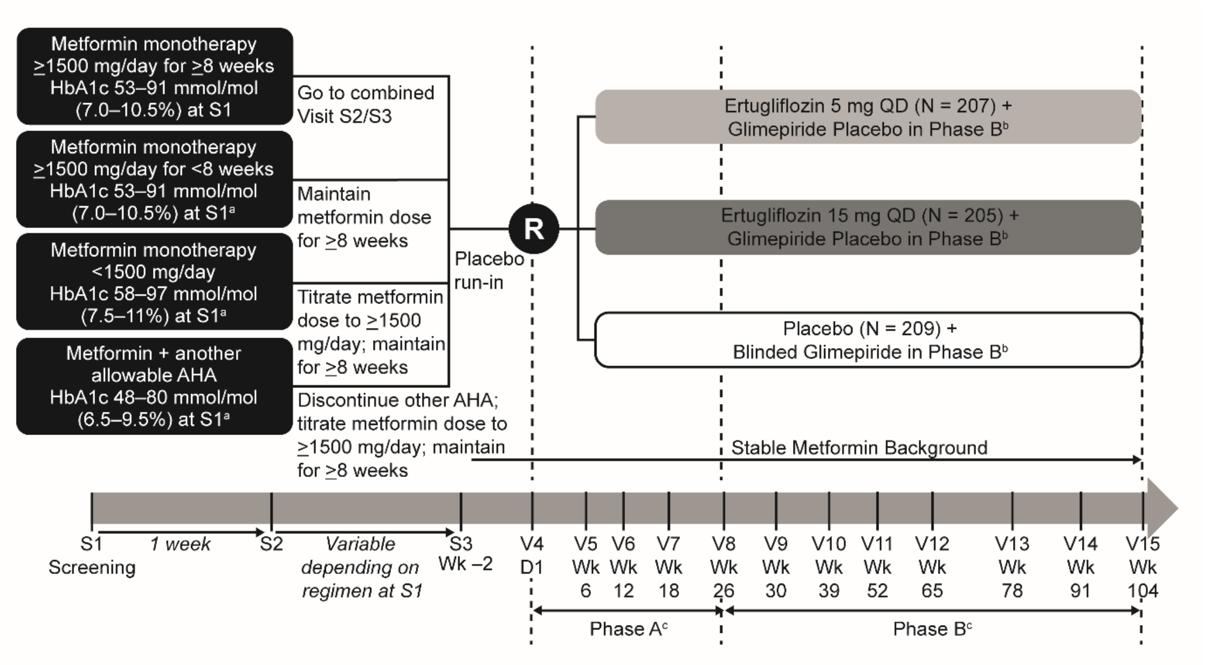

[a]Participants were randomised only if HbA_{1c} at S3 was 53–91 mmol/mol (7.0–10.5%). [b]Blinded glimepiride/glimepiride placebo was only for participants not receiving glycaemic rescue in Phase A and whose fasting fingerstick glucose was ≥6.1 mmol/l (≥110 mg/dl). [c]Glycaemic rescue therapy (open-label glimepiride) and open-label basal insulin initiated for participants who met progressively more stringent glycaemic thresholds in Phase A; basal insulin also initiated in Phase B for participants who met glycaemic thresholds.

AHA, anti-hyperglycaemic agent; D, day; N, number of randomised participants in each treatment group; R, randomisation; S, screening; V, visit; Wk, Week

Figure from Gallo et al. 2019 under the terms of the Creative Commons Attribution-NonCommercial-NoDerivatives 4.0 International license (https://creativecommons.org/licenses/by-nc-nd/4.0/). The following changes have been made to the figure: superscripts in letters (originally in symbols), depiction of the HbA_{1c} inclusion criterion in mmol/mol, the display of some text on additional lines and changes to the figure legend text (use of glycaemic instead of glycemic and addition of VERTIS in the abbreviations).

ESM Fig. 2 VERTIS SU study design

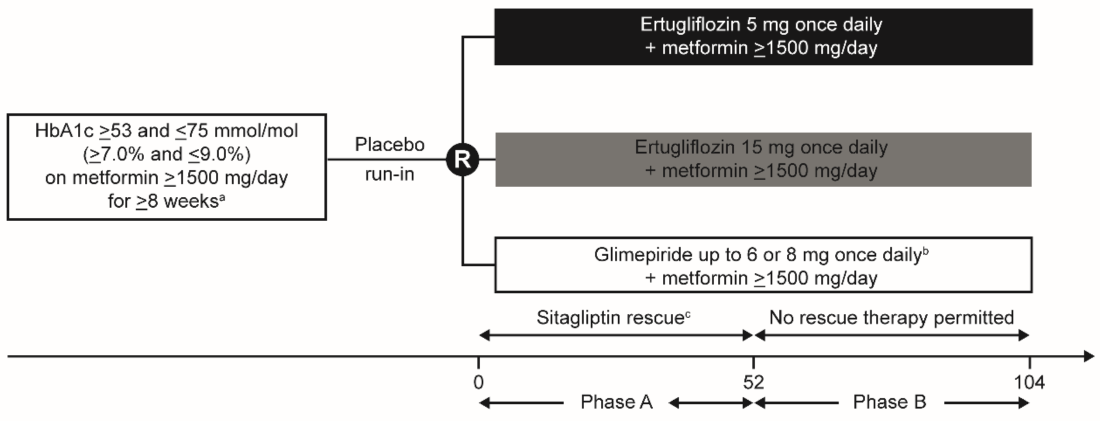

X-axis values are weeks. [a]Patients on one of the following regimens were also eligible to enter the screening period and could enroll in the study if they met entry criteria after the wash-off/dose titration/stabilisation period: on metformin monotherapy ≥1500 mg/day <8 weeks, HbA_{1c} ≥53 mmol/mol and ≤75 mmol/mol (≥7.0% and ≤9.0%) – patients were to maintain metformin dose ≥1500 mg/day for ≥8 weeks; on metformin monotherapy <1500 mg/day and with HbA_{1c} ≥58 mmol/mol and ≤80 mmol/mol (≥7.5% and ≤9.5%) – patients were titrated to metformin ≥1500 mg/day and were to maintain metformin dose for ≥8 weeks; on metformin + single allowable antihyperglycaemic agent[d] and HbA_{1c} ≥48 mmol/mol and ≤69 mmol/mol (≥6.5% and ≤8.5%) – patients were to discontinue non-metformin AHA, titrate metformin to ≥1500 mg/day (if necessary), and maintain metformin dose ≥1500 mg/day for ≥8 weeks (≥10 weeks for patients discontinuing SU therapy). [b]Glimepiride was initiated at 1 mg once daily and up-titrated to a maximum of 6 or 8 mg/day according to the local label or maximum tolerated dose. [c]Patients rescued with sitagliptin in Phase A were not eligible to enter Phase B; patients were not rescued during Phase B. [d]This included SUs at <50% of the maximum approved dose in the local country label, dipeptidyl peptidase-4 inhibitors, meglitinides or alpha glucosidase inhibitors.

AHA, antihyperglycaemic agent; SU, sulfonylurea; R, randomisation

Figure from Hollander et al. 2019 under the terms of the Creative Commons Attribution-NonCommercial-NoDerivatives 4.0 International license (https://creativecommons.org/licenses/by-nc-nd/4.0/). The following changes have been made to the figure: text in normal font (originally bold font was used) and changes to the figure legend text (use of antihyperglycaemic instead of antihyperglycemic and addition of VERTIS in the abbreviations).
